# Understanding attitude, practices and knowledge of zoonotic infectious disease risks among poultry farmers in Ghana

**DOI:** 10.1002/vms3.257

**Published:** 2020-04-03

**Authors:** Matilda Ayim‐Akonor, Ralf Krumkamp, Jürgen May, Eva Mertens

**Affiliations:** ^1^ Department of Infectious Disease Epidemiology Bernhard Nocht Institute for Tropical Medicine Hamburg Germany; ^2^ Department of Animal Health and Food safety Council for Scientific and Industrial Research‐Animal Research Institute Accra Ghana

**Keywords:** attitudes, farmers, Ghana, health knowledge, practices, zoonoses

## Abstract

Zoonotic infectious diseases (ZIDs) are increasing globally, and livestock farmers in low‐ and middle‐income countries are at particularly high risk. An evaluation of farmer's behaviour on farms can be used to identify the risk factors and to develop tailored control strategies. This study documents the knowledge of zoonotic poultry diseases (ZPD) among 152 poultry farm workers (respondents) from 76 farms in the Ashanti region of Ghana and assessed their on‐farm attitude and practices that increase their risk to exposure of ZPD. The median age of respondents was 29 years, 91.4% (*n* = 139) had a formal education, and 80.9% (*n* = 123) had worked on the farm for more than 1 year. The majority of farms (*n* = 69, 90.8%) had multiple flocks and 27.6% (*n* = 21) kept other animals, of which 57.1% (*n* = 12) were pigs. The majority of respondents had good knowledge about poultry diseases but not about ZPD. A higher level of education and longer work experience improved respondents’ knowledge of poultry and ZPD. Although respondents identified the wearing of personal protective equipment (PPE) as a major ZPD preventive measure, the majority did not put that knowledge into practice. Most farms (71.1%, *n* = 54) had no footbath and 55.3% (*n* = 42) deposited farm‐waste on the farm. While 97.4% (*n* = 148) of respondents washed their hands after working, only 48.7% (*n* = 74) wore protective footwear, 2.7% (*n* = 4) wore overalls, 2% (*n* = 3) wore nose masks and none (*n* = 0) wore gloves. The husbandry practices and attitude of farmers expose them to pathogens on the farm and increase their risk of becoming infected with ZPD in the sub‐region. The results from this study could be used to promote human health among farm workers in Ghana.

## Graphical abstract

Poultry farmers in Ghana have low knowledge level about zoonotic poultry diseases. Their attitude and practices on farms increase their risk of becoming infected with zoonotic poultry diseases

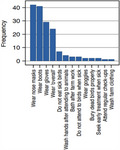

## 1 INTRODUCTION

The global prevalence of human infectious diseases remains high and zoonotic infectious diseases (ZIDs) form the highest percentage, accounting for 61% of all known infectious diseases, and 75% of emerging infectious diseases (WHO, 2011). ZIDs are also of agricultural and economic importance, as they impact animal health, reduce productivity, affect income and food security of farm products. The revenue loss from the imposition of trade restrictions, low patronage by consumers and increased marketing costs to regain consumer confidence may impede economic growth of countries, where ZIDs are common in farmed livestock (Halliday et al., 2015; McDermott & Arimi, 2002; WHO, 2006). There has been a growing demand for animal products in many urban and peri‐urban communities in low‐ and middle‐income countries (LMIC) due to increasing rural–urban migration, and changes in socio‐cultural and socio‐economic status. This has contributed to intensification of livestock production in densely populated areas, thus increasing the risk of human infections with zoonotic pathogens (Thornton, 2010; Zinsstag et al., 2007). Livestock farmers remain at high risk of acquiring ZIDs due to their proximity and frequent contact with the animals and their environment.

Thirty percent of livestock farmers from developing countries live in sub‐Saharan Africa (Thornton et al., 2002). Their husbandry practices are often based on traditional knowledge and skills inherited from their ancestors, which may be fused with modern methods of livestock keeping. Such practices may differ between and within countries even for the same species and therefore the potential risk of becoming infected with ZIDs may differ (FAO, 2009; Mangesho et al., 2017). Poultry production is a major component of the livestock sector in Ghana and contributes substantially to the animal protein source and food security. Several poultry diseases with both zoonotic and non‐zoonotic potential characterise the industry (Andoh et al., 2016; Ayim‐Akonor, Obiri‐Danso, Toah‐Akonor, & Sellers, 2018; FAO, 2014). Three outbreaks of highly pathogenic avian influenza virus (HPAIV) H5 have affected the Ghanaian poultry industry in the last 12 years (Asante et al., 2016; OIE, 2019). Although no human infections occurred, the risk to poultry farmers remains high; 16 countries worldwide have recorded human infections with a 53% fatality rate and contact with infected poultry or the environment was identified as transmission pathways (WHO, 2019).

In this study, we aimed to assess the knowledge level of poultry farmers regarding zoonotic poultry disease (ZPD) and further evaluate their on‐farm attitude and practices that increase their risk of becoming infected with ZPD. The information will provide baseline data to develop practical control methods to reduce zoonotic transmission among poultry farmers in sub‐Saharan Africa.

## 2 MATERIALS AND METHODS

### 2.1 Study area

The study was performed in the Ashanti region of Ghana from April 2016 to February 2017. The Ashanti region is located in the forest belt of the country. It is the third largest region covering 10.2% of the total country land size. The region has the highest human population (19.4% of national population). The Ashanti region is the second largest poultry‐producing region, holding 28% of the total poultry population in Ghana. Breeders, layers (egg‐type) and broilers (meat‐type) form the bulk of poultry kept by farmers, with layer birds dominating the sector (FAO, 2014; Ghana Ministry of Food & Agriculture, 2015; Nyanteng et al., 2013).

### 2.2 Ethical consideration

Ethical approval for the study was obtained from the ethics committees of the Council for Scientific and Industrial Research, Ghana (RPN 001/CSIR‐IACUC/2016) and the Ethik‐Kommission der Ärztekammer Hamburg (PV5296) in Germany.

### 2.3 Farm selection

Members of the regional poultry farmer association were contacted personally or through a mobile phone call. The study was explained to the farmers and, where informed consent was provided, farms were visited to conduct interviews. Famers were asked whether they knew colleagues who were not members of the poultry farmer association, and these were considered as potential study participants (snow‐ball sampling). Questionnaires were administered only on farms if birds were present and at least one farm worker worked at the time of visit.

### 2.4 Questionnaire administration

A questionnaire with open‐ended and close‐ended questions was used. Questionnaires were administered in English and in the local language ‘Twi’ (responses were translated into English for analysis). The questionnaire included sections on farm characteristics, farm husbandry practices, demographics, biosecurity practices, knowledge of poultry diseases, awareness of zoonotic diseases and self‐protection from zoonotic diseases.

### 2.5 Data entry and analysis

Medians and interquartile ranges were computed for continuous variables, and the frequency and percentages were computed for categorical variables. Data on age, education and length of employment on the farm were dichotomised to calculate association measures. A dichotomised knowledge level score of poultry diseases (good or poor) was developed based on the respondents' ability to name at least one correct visible clinical sign indicating animal disease and being able to name at least one poultry disease and its corresponding clinical signs. A dichotomised knowledge level score of ZPDs (good or poor) was developed based on the respondents' awareness of becoming infected with certain diseases of poultry, correctly naming at least one zoonotic poultry disease, and mentioning at least one method to protect against zoonotic poultry disease. Risk ratios (RR), with their corresponding 95% confidence intervals (CI), were calculated to estimate associations between the dichotomised scores and individual characteristics. A binomial regression model, with a log‐link function, was fitted to calculate multivariate models. Backward elimination was applied to select the final models. All analyses were conducted using the statistical program Stata (Version 14, StataCorp).

## 3 RESULTS

### 3.1 Farm characteristics

In all, 76 poultry farms in the study region were visited during the sampling period. Seven farms were reported to have one flock (9.2%), 38 (50%) to have two or three flocks and 31 (40.8%) to have more than three flocks. The total number of birds on the farms varied from 50 to more than 10,000. In all, 55 farms had less than 5,000 birds (72.4%), 13 (17.1%) had 5,000–10,000 birds and eight (10.5%) had more than 10,000 birds. Most farms (*n* = 55, 72.4%) kept only chickens. Some farms (*n* = 21, 27.6%) additionally kept other animals, predominantly pigs (*n* = 12, 57.1%), ruminants (*n* = 7, 33.3%) and others such as free‐range chicken, ducks, guinea fowl and turkeys. The majority of farms (*n* = 65, 85.5%) prepared their animal feed at local feed mills.

Different preventive measures against poultry diseases were in place. Few farms (*n* = 22, 28.9%) had footbaths, of which 12 (54.5%) treated with fresh disinfectants weekly and 10 (45.5%) applied fresh disinfectants occasionally. Water troughs were washed daily on all farms. Wood shavings were used as bedding materials by nearly all farms (*n* = 74, 97.4%) and were changed occasionally (*n* = 28, 36.8%) or at the end of the production cycle (*n* = 48, 63.2%). Nearly half of the farms (*n* = 34, 44.7%) disposed their farm waste outside the farm premises. All farms (*n* = 76, 100%) vaccinated their poultry against Newcastle disease virus (NDV) and infectious bursal disease virus (causing a disease also known as Gumboro). In addition, layer and breeder farms (*n* = 68, 89.5%) vaccinated against fowl poxvirus. Different personnel carried out vaccination and treatment of sick birds. Veterinarians administered vaccines on half of the farms (*n* = 39, 51.3%), farm personnel on 34 (44.7%) farms and both veterinarian and farm personnel on fewer (*n* = 3, 3.9%) farms. Veterinarians treated sick animals on the majority (*n* = 47, 61.8%) of farms; some farms (*n* = 21, 27.6%) self‐medicated and the minority (*n* = 8, 10.5%) practiced both. Almost all farms (*n* = 75, 98.7%) sold their birds live at the farm gate. Most farms (*n* = 45, 59.2%) had two or three employees.

### 3.2 Demographics

A total of 152 respondents participated in the study. Of these, 131 (86.2%) were males. The majority (*n* = 65, 42.8%) were 20–29 years with a median age of 29 years (IQR = 23.0–41.6). Most respondents (*n* = 57, 37.5%) had middle‐school level education and few (*n* = 13, 8.6%) had non‐formal education. Almost half (*n* = 75, 49.3%) of respondents had worked on their farm for 1–5 years (Table 1).

**TABLE 1:** Demographic characteristics of respondents

Variable	*N* (%)
Female sex	21 (13.8)
Age (years)	
<20	12 (7.9)
20–29	65 (42.8)
30–39	34 (22.4)
40–49	19 (12.5)
50–59	16 (10.5)
60–69	4 (2.6)
>70	2 (1.3)
Education level	
Primary	11 (7.2)
Middle school	57 (37.5)
Senior high school	30 (19.7)
Tertiary	41 (27.0)
None	13 (8.6)
Length of employment at present farm	
<1 yr	29 (19.1)
1–5 yrs	75 (49.3)
>5 yrs	48 (31.6)

### 3.3 Safety and hygiene practices of respondents on the farm

Respondents reported on various safety and hygiene practices that they perform routinely on their farms. Most respondents (*n* = 148, 97.4%) changed their clothes before starting work on the farm. Few respondents (*n* = 4, 2.7%) wore ‘overalls’ and nearly half (*n* = 74, 48.7%) wore footwear that covers the entire foot. Most respondents (*n* = 151, 99.3%) changed their footwear before leaving the farm. The majority of respondents (*n* = 149, 98%) did not wear nose masks and none (*n* = 152, 100%) wore gloves when working on the farm. However, nearly all respondents (*n* = 148, 97.4%) washed their hands before leaving the farm (Table 2).

**TABLE 2:** Personal protective equipment usage and hygiene practices among respondents

Parameter	*N* (%)
Change clothes before attending to poultry	148 (97.4)
Type of clothes worn to attend to poultry	
Overall	4 (2.7)
Own clothes	144 (97.3)
Change clothes before exiting farm	148 (100)
Wear protective footwear	74 (48.7)
Change footwear before leaving the farm	151 (99.3)
Wear nose mask	3 (2.0)
Wear gloves	0 (0.0)
Wash hands before leaving farm	148 (97.4)

### 3.4 Knowledge of poultry diseases

**FIGURE 1:**
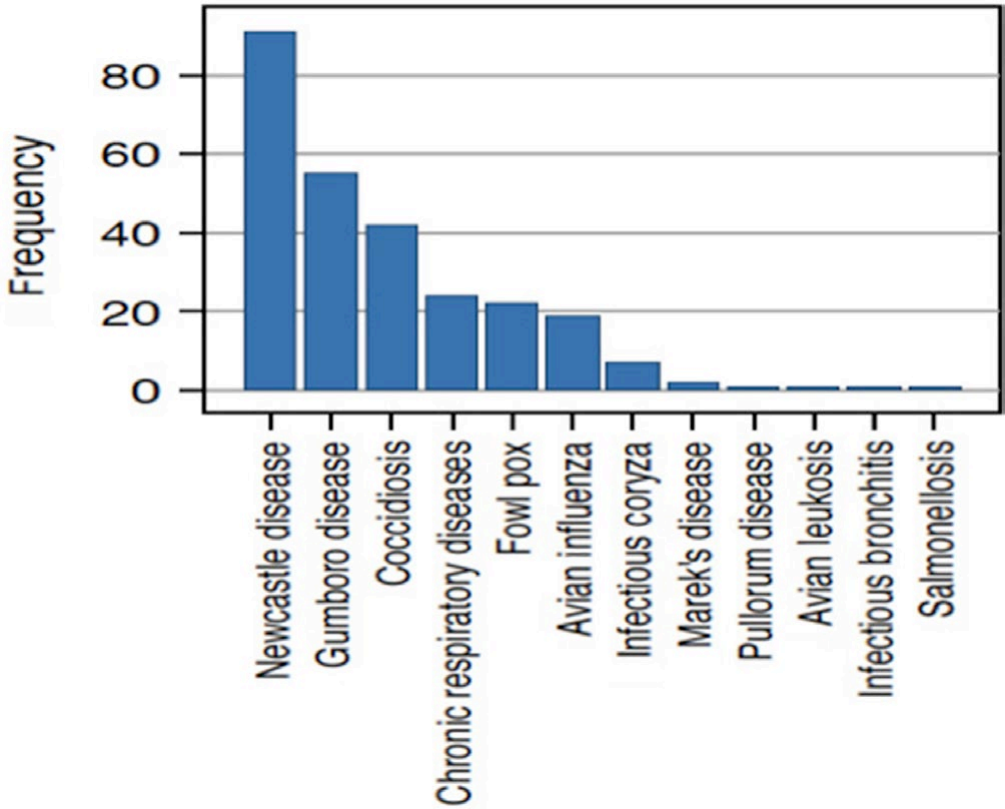
Poultry diseases named by respondents (*n* = 266)

Most respondents (*n* = 132, 86.8%) could identify when their birds were sick. Respondents used clinical signs exhibited by their chickens to determine their health status. Common clinical signs reported comprised the following: greenish diarrhoea, weakness, loss of appetite, trachea rales, cough, sneeze, drop in egg production, bloody spots in faeces, pox on comb and ruffled feathers. Of the respondents who could identify sick animals by clinical signs, very few (*n* = 29, 22%) could not name any poultry disease. The majority of respondents (*n* = 101, 76.5%) correctly named at least one poultry disease with one or more associated clinical sign(s). In total, respondents named 12 different poultry diseases (Figure 1). NDV, Gumboro disease and Coccidiosis were most frequently named while infectious bronchitis and salmonellosis were the least frequently mentioned (Figure 1).

### 3.5 Awareness and self‐protection from zoonotic poultry diseases

**FIGURE 2:**
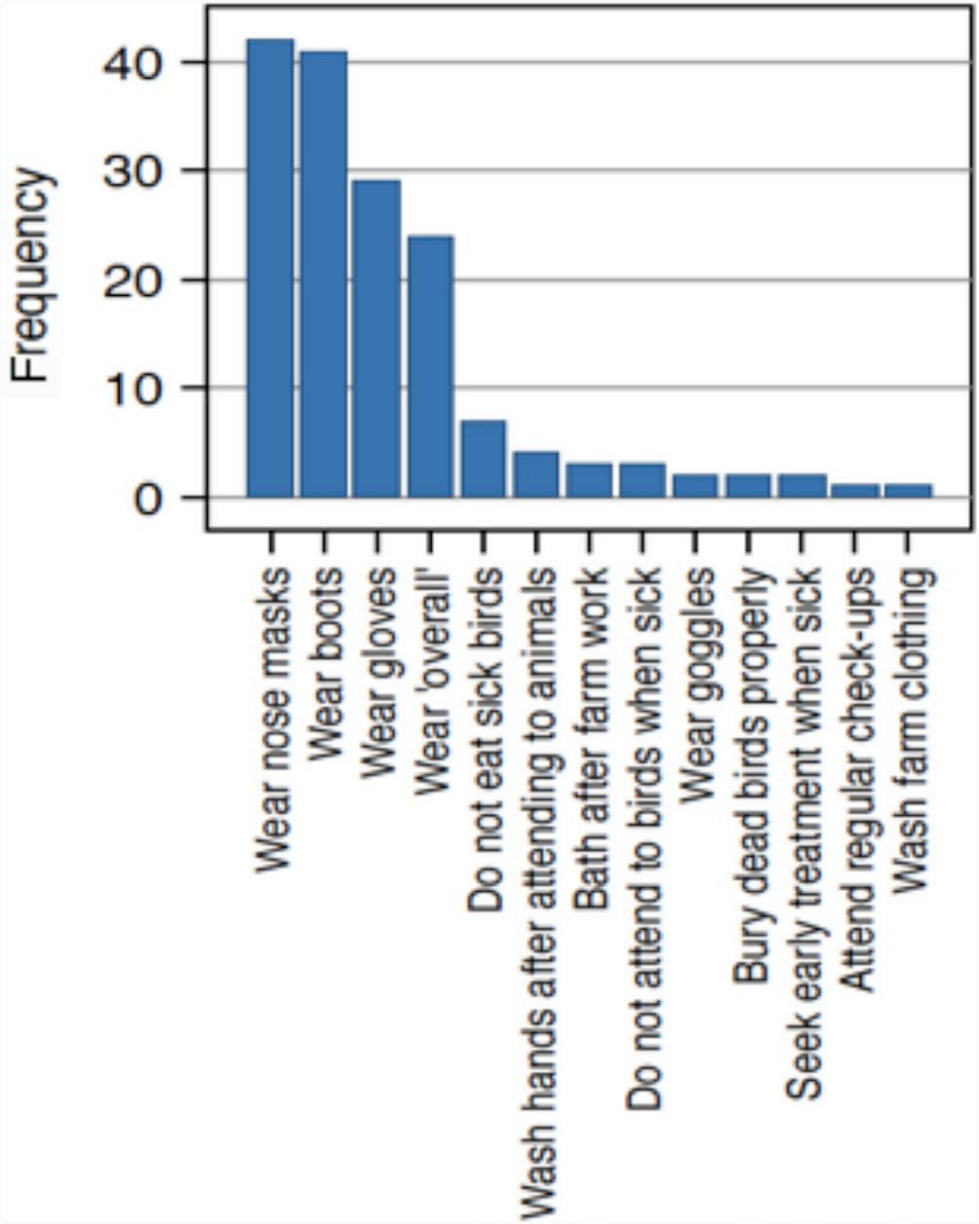
Zoonotic preventive measures named (*n* = 161)

In all, 87 (57.2%) respondents were aware that they could become infected with certain poultry diseases. Of those, nearly half (*n* = 39, 44.8%) could name at least one zoonotic poultry disease. Respondents named avian influenza (AI), NDV and salmonellosis as diseases they could contract from their poultry. AI was the most frequently named (74.5%) while NDV and salmonellosis accounted for 15.7% and 9.8%, respectively. Respondents could name 13 different ways to protect themselves from becoming infected with pathogens from poultry. The wearing of nose mask, wellington boots, gloves and overalls while working was the most frequently used zoonotic preventive method. Avoiding the consumption of sick birds, proper disposal of dead birds and regular washing of farm clothing were less frequently mentioned (Figure 2).

The majority of respondents (*n* = 88, 57.9%) had good knowledge about poultry diseases. The age, educational level and experience of respondents influenced their knowledge level score on poultry diseases. Respondents older than 29 years of age were 60% more likely to have good knowledge of poultry diseases than respondents 29 years and below (RR = 1.6, 95% CI = 1.2–2.1). Respondents with a higher education level were about twice as likely to have good knowledge about poultry diseases than respondents with lower education levels (RR = 1.8, 95% CI = 1.4–2.4). Respondents with more than 5 years of employment on the farm were 50% more likely to have good knowledge about poultry diseases than respondents who have spent 5 years or less on the farm (RR = 1.5, 95% CI = 1.2–1.9). In binomial regression, estimates comparable to the crude results were calculated (Table 3).

**TABLE 3:** Factors influencing respondents' knowledge of poultry diseases

Parameter	High level *N* (%)	Crude RR		Regression model	
		RR	95% CI	aRR	95% CI
Sex					
Female	9 (42.9)	Ref.	0.4–1.2		
Male	79 (60.3)	0.7			
Age (years)					
Up to 29	35 (45.5)	Ref.	1.2–2.1		
>29	53 (70.7)	1.6			
Education level					
Low level	34 (42.0)	Ref.	1.4–2.4	1.7	1.3–2.3
High level	54 (76.1)	1.8			
Duration of employment on farm (years)					
Up to 5	52 (50.0)	Ref.	1.2–1.9	1.4	1.1–1.7
>5	36 (75.0)	1.5			

Abbreviations: aRR, adjusted risk ratio; CI, confidence interval; RR, risk ratio.

The knowledge level of respondents on ZPD varied considerably from their knowledge of poultry diseases. A quarter (*n* = 38, 25%) of respondents had good knowledge about ZPD. Respondents with a higher education level were 10 times more likely to have good knowledge of ZPD than respondents with a lower education level (RR = 9.7, 95% CI = 3.6–26.0). Respondents, who worked on the farm for >5 years, were about twice as likely to have good knowledge of ZPD than respondents with up to 5 years of employment experience (RR = 2.0, 95% CI = 1.1–3.3). The binary regression yields comparable results as the crude estimates, highlighting that the chosen variables were unconfounded (Table 4).

**TABLE 4:** Factors influencing respondents' knowledge of zoonotic poultry diseases

Parameter	Good level *N* (%)	Crude RR		Regression model	
		RR	95% CI	aRR	95% CI
Sex					
Female	5 (23.8)	Ref	0.4–2.2		
Male	33 (25.2)	1.0			
Age (years)					
Up to 29	10 (13.0)	Ref	1.5–5.5		
>29	28 (37.3)	2.9			
Education level					
Low level	4 (5.6)	Ref	3.6–26.0	9.6	3.6–25.5
High level	34 (47.9)	9.7			
Duration of employment on farm (years)					
Up to 5	20 (19.2)	Ref	1.1–3.3	1.9	1.2–2.9
>5	18 (37.5)	2.0			

Abbreviations: aRR, Adjusted risk ratio; CI, confidence interval; RR, risk ratio.

## 4 DISCUSSION

Proper biosecurity measures (i.e. the implementation of measures that reduce the risk of the introduction and spread of disease agents, FAO/OIE/World Bank, 2008) when adequately practiced on the farm can reduce the risk of introduction and spread of pathogens on farms and further reduce risk of transmission to farmers (Nyaga, 2007). Farmers in Ghana set and operate their own biosecurity standards based largely on their own experience (Aning, Turkson, & Asuming‐Brempong, 2009). Our study showed that farms did not comply with all the recommended biosecurity practices and may therefore be at higher risk of outbreaks of infectious diseases on farms and possible ZID spread to humans.

The majority of farmers (81%) have been on the same poultry farm for over 1 year, yet the adoption of on‐farm disease mitigating measures like cleaning and disinfection was low. Farmers practiced multi‐species (especially chickens and pigs) and multi‐age farming (multiple flock of different ages) without the use of footbaths. This does not provide sufficient disinfection of housing and zero fallow period to reduce microbial load in the poultry house. The practice of multi‐species provides an enabling environment for generating re‐assortment of influenza viruses with zoonotic and pandemic potential from influenza viruses that may be circulating among poultry and pigs in the region (Adeola, Olugasa, & Emikpe, 2015, 2016). Generated waste (including bedding materials of wood shavings together with poultry faeces, feathers, feed and farm dust) is deposited largely on the farm premises which may contaminate the farm environment with potential microbes of economic, environmental and public health importance such as Salmonella sp. and AIV (Andoh et al., 2016; Stephens & Spackman, 2017; Vadari, Mason, & Doerner, 2006; WHO, 2006).

Farms retailed their live birds at the farm gate. This practice brings retailers onto the farm premises regularly exposing them to pathogens circulating on the farm and its environs. The practice also introduces pathogens from carriages of the retailers such as vehicles and cages, onto the farm premises. Aning et al. (2009) observed that public transport is mostly used to move birds in Ghana and that these vehicles are not adequately disinfected before and after being used to transport the birds, posing a public health risk. The unregulated movement of live birds in the country (which is prohibited only during AI outbreaks) further aid the spread of infectious pathogens within and/or between regions in the country and cause exposure risk of the public to airborne zoonotic pathogens such as low pathogenic AI that the birds may be shedding without demonstrating obvious clinical signs.

The wearing of appropriate PPE and adequate farm hygiene practices by farm workers reduces their risk of exposure to occupational health hazards (European Commission, 2012). The majority of respondents have worked on their poultry farms for over 1 year and may have been exposed to infected poultry and the contaminated environment of the farm. Respondents washed their hands regularly but did not utilise PPE for farm activities such as vaccinations and treatment of sick birds. Although washing of hands is a good hygiene practice, it is the use of detergents such as soap in hand washing that is effective in reducing risk of infection significantly. We did not explore the use of detergents by respondents and can therefore not determine the reduced risk level of hand washing. Respondents did not wear nose masks and remain at high risk of airborne transmitted ZIDs such as AI if circulating in the farms (Harder, Buda, Hengel, Beer, & Mettenleiter, 2016). Gloves were not worn and farm clothing was predominantly a separate set of clothes (often a T‐shirt and a pair of shorts or trousers) that the farmer kept on the farm. The use of these separate clothing does not provide the same level of protection as would be provided by overalls (Odo et al., 2015). Interestingly, the majority of respondents had formal education and largely recognised that wearing of PPE is an important preventive measure for ZIDs yet did not implement their use. According to the FAO (2014), poultry farmers in Ghana received extensive training on biosecurity and biosafety practices after the original AI outbreak. However, this study reveals poor adoption and implementation among respondents, implying that education and awareness alone may not be enough to bring about behavioural change among farmers. Behavioural change among respondents may require a multidisciplinary approach including communication and economic analysis. The poor adoption of PPE we observe here is similar to that reported in Bangladesh and Thailand (Odo et al., 2015; Sarker, Sumon, Khan, & Islam, 2016).

Infectious diseases are of major concern to the global poultry industry as frequent outbreaks reduce net profit margins. Our respondents had good knowledge about poultry diseases, particularly those that are endemic and have major economic importance in the country (FAO, 2014). In the multivariate analysis, this good knowledge of poultry diseases was predicted by long years of employment on the farm and having a higher level of education. This good veterinary knowledge and associated predictive factors did not influence respondents’ preventive practices on the farm showing knowledge gaps of farm husbandry practices and disease mitigation. This observation is contrary to that reported in China where good veterinary knowledge coupled with longer farming experience of respondents correlated with higher adoption and implementation of disease preventive practices (Huang, Zeng, & Wang, 2016).

As respondents stay longer on the farm, they gain experience in raising the animals and are better able to recognise and treat diseases. This may influence their choice of health care assistance when needed. A study by Turkson (2009) shows that farmers in Ghana rely on their own experience and that of their colleague farmers to buy and dispense drugs to their animals rather than to seek professional assistance. However, we observed skewness towards veterinarians for both disease treatment and vaccination services despite respondents’ good knowledge of poultry diseases. This observation agrees with recent report on the use of antibiotics in the poultry industry in the same region (Boamah, Agyare, Odoi, & Dalsgaard, 2016).

The majority (*n* = 114, 75%) of respondents did not have good knowledge about ZPD according to our score. For the few who had, their good knowledge score was predicted by higher education level and long employment on the farm, similar to that reported in China and Italy on AIV (Abbate, Di Giuseppe, Marinelli, & Angelillo, 2006; Chen et al., 2015). Our respondents were predominantly aware of the zoonotic potential of AIV and to a lesser extent, NCDV and the foodborne pathogen Salmonella. However, they were unaware to the zoonotic potentials of Avian chlamydiosis that cause flu‐like symptoms among others, and other foodborne pathogens such as Campylobacter (Andoh et al., 2017; European Commission, 2002; Fraser, Williams, Powell, & Cook, 2010; Osei‐Tutu & Anto, 2016).

The limited knowledge of farmers about ZPD may account for their relatively poor attitude towards the wearing of PPE. Farmers were unaware of the zoonotic risk of certain diseases from the animals they keep and the health implications thereof. Our study did not directly assess the knowledge of the transmission route of ZPD among the farmers. However, we identified an implementation gap in which respondents are aware of preventive methods against ZPD but do not put them into practice.

Poultry farmers in the Ashanti region of Ghana have a good knowledge of poultry diseases, which may cause them to treat their birds when sick rather than seek professional help. However, their understanding of becoming infected with specific pathogens from their poultry is low. Farmers’ husbandry practices and attitude are not enough to prevent infections and or reduce spread on the farm, thereby increasing their risk of becoming infected with ZPD. The reason(s) for the poor adoption and implementation of biosecurity and biosafety measures among the farmers despite their awareness of these measures should be explored and appropriate interventions instituted.

## CONFLICT OF INTEREST

Authors and funders declare no conflict of interest.
